# The impact of the Covid-19 pandemic on the effectiveness of psychosomatic rehabilitation in Germany

**DOI:** 10.1186/s12913-024-11170-1

**Published:** 2024-06-11

**Authors:** Klaus Kaier, Jakob Knecht, Lukas Nalbach, Mirjam Körner

**Affiliations:** 1 Institute of Medical Biometry and Statistics, Faculty of Medicine and Medical Center, University of Freiburg, Hugstetter Str. 49, 79106 Germany, Freiburg, Germany; 2 Medical Psychology and Medical Sociology, Faculty of Medicine and Medical Center, University of Freiburg, Freiburg, Germany

**Keywords:** Covid-19 pandemic, Psychosomatic rehabilitation, HEALTH-49, ICF AT-50 Psych, PAREMO, SIMBO-C

## Abstract

**Background:**

The aim of the present study is to investigate the impact of the Covid-19 pandemic on the effectiveness of psychosomatic rehabilitation.

**Methods:**

Between April 2019 and March 2022, a total of 18,388 patients from 7 rehabilitation centres could be included in the study. For each patient, score values from the HEALTH-49 and ICF AT-50 Psych questionnaires were calculated at the beginning and at the end of rehabilitation and the effectiveness of the rehabilitation program was determined by comparing the scores at the beginning and at the end of the rehabilitation programme. Using risk adjusted linear mixed models, three time intervals were compared: a pre-pandemic episode (April 2019 to March 2020), the first year of the pandemic (April 2020 to March 2021) and the second year of the pandemic (April 2021 to March 2022).

**Results:**

Overall, it can be stated that the pandemic has substantially impaired the effectiveness of psychosomatic rehabilitation measures. This phenomenon can be observed across a wide range of psychosocial markers and even two years after the start of the pandemic there is no end to the limited effectiveness. With regard to ‘psychological and somatoform disorders’, for example, there was a relative decrease in the effectiveness of the rehabilitation measure by 11.29% in the first year of the pandemic compared to the pre-pandemic episode, *p* < 0.001. In the second year of the pandemic, the effectiveness of the rehabilitation measure was still decreased by 8.8% compared to the pre-pandemic episode, *p* < 0.001. In addition, the evaluations show that a division of the pandemic effect into direct effects (on the individual) and indirect effects (via further complication of the occupational problem environment) can be made and that the pandemic-related complication of the occupational problem environment are still prevalent more than two years after the start of the pandemic.

**Discussion:**

The Covid-19 pandemic has had a significant impact on the psychosomatic rehabilitation programs reducing the effectiveness of treatment not only for a short period of time but constantly until March 2022.

**Trial Registration Number:**

DRKS00029669; Date of registration: 02/08/2022.

**Supplementary Information:**

The online version contains supplementary material available at 10.1186/s12913-024-11170-1.

## Introduction

A disaster is defined as “a potentially traumatic event that is collectively experienced, has an acute onset, is time-delimited and may be attributed to natural, technological and human causes” [1]. By this definition, the global pandemic can be considered a major disaster. Changes in the work environment, restrictions on leisure activities and social interactions had an enormous impact on the daily lives of people around the world, accompanied by uncertainty about the duration, severity and possible long-term effects.

A number of studies have shown that mental disorders, such as depression, are more common and tend to worsen in the aftermath of major disasters. A systematic review on the topic report higher rates of psychological distress and higher rates of psychiatric disorders after natural disasters in exposed groups compared with unexposed controls, as well as compared with pre-disaster surveys [2]. Similar findings were reported after the economic recession in Europe between 2008 and 2013 [3].

Another systematic review describes the prevalence of depression, anxiety and stress in the general population at the onset of the Covid-19 pandemic [4]. They reported a prevalence of 33.7% for depression, 31.9% for anxiety and 29.6% for stress. Similar results were reported by the Swiss Corona Stress Study [5]. Within the general population, studies have identified more affected groups and several risk factors associated with a higher risk of mental disorders. Female sex, younger age and lower levels of education had a negative impact on mental well-being during the pandemic [6–8]. In addition, people with pre-existing mental disorders are more vulnerable to the increased exposure to stressors during the pandemic, which may lead to a worsening of psychiatric symptoms [9–11].

There are about 25,000 beds in psychosomatic rehabilitation centres in Germany, treating about 5/1,000 of the working age population per year [12]. The aim is to prevent and treat chronic illnesses by reducing symptoms, improving the patient’s own abilities, self-efficacy and quality of life, and reintegrating the patient into a job that meets his or her needs and limitations. During a stay of approximately 5 weeks, patients are treated with a multi-dimensional method including patient education, medication, psychotherapy, occupational and sports therapy, and the help of social workers. Usually, the rehabilitation is provided as an inpatient program by the statutory pension insurance. After their discharge, the effectiveness of the process is evaluated by an expert, including an assessment of the financial benefit to the pension fund and the improvement in the patient’s medical and psychological status.

During the pandemic, it was impossible to maintain the usual number of patients in rehabilitation hospitals, which led to a 21.8% decrease in the use of psychosomatic rehabilitation between March and December 2020 [13]. In addition, the regular daily routine and interaction between patients had to be adapted to the current regulations, which varied from region to region. Some general safety precautions were introduced, such as testing patients for Covid-19 on arrival, wearing medical face masks, serving meals in the patient’s room or only in small groups, videoconferencing lectures instead of face-to-face meetings, and reducing the size of groups in group therapy. In addition, visits were strictly regulated or banned altogether.

Under these circumstances, it can be assumed that not only the pandemic itself, but also the various restrictive measures had an impact on the condition of the patients before they started rehabilitation, as well as on the effectiveness of the rehabilitation.

Therefore, this study uses data from 7 rehabilitation centres in Germany to address the following research questions:

1. What are the pandemic-related differences in the mental health of patients at the beginning of rehabilitation?
2. Do the different waves of the pandemic have an impact on the effectiveness of the rehabilitation programme?
3. Is it possible to distinguish between direct effects of the pandemic on the individual’s mental well-being and indirect effects via complication of the occupational problem?

## Methods

Continuous and categorical variables were compared using Kruskal-Wallis and chi-square tests. Primary success factors of psychosomatic rehabilitation were responses to the HEALTH-49 questionnaire. The HEALTH-49 questionnaire is a widely used instrument for assessing health-related quality of life in patients undergoing psychosomatic rehabilitation [14, 15]. In detail, the following domains of the HEALTH-49 questionnaire were used: ‘Psychological and somatoform disorders’, ‘Psychological well-being’, ‘Interactional problems’, ‘Self-efficacy’, ‘Activity and participation’, ‘Social support’ and ‘Social stress’.

Furthermore, the domain ‘Activity and Participation’ was also collected using the International Classification of Functioning, Disability and Health (ICF) questionnaire [16]. The ICF AT-50 Psych is a self-assessment questionnaire based on the ICF for mapping activity and participation of people with mental disorders. With a total of 50 items, activity (verbal competence, fulfilment of requirements, fitness and well-being) and participation (social relationships and activities, closeness in social relationships, social consideration) are assessed each with three scales [17–19]. The rationale for choosing the HEALTH-49 and ICF-AT 50 domains as indicators of rehabilitation effectiveness lies in their comprehensive and standardized approach to assessing health outcomes. These tools are grounded in the International Classification of Functioning, Disability, and Health (ICF) framework, which offers a holistic view of health by considering both physical and psychosocial dimensions.

Both HEALTH-49 and ICF AT 50 Psych scores were calculated at the beginning and at the end of rehabilitation and the effectiveness of the rehabilitation program was determined by comparing the scores at the beginning and at the end of the rehabilitation programme. In order to assess the participant’s willingness to change, the respective domain of the PAREMO questionnaire (patient questionnaire of rehabilitation motivation), was assessed at the beginning of the rehabilitation program [20]. In order to assess participants’ occupational problems and the job situation, the SIMBO-C score was calculated at the beginning of the rehabilitation program [21, 22].

For the analysis of the Covid-19 pandemic, the data were divided into three episodes. A pre-pandemic episode (April 2019 to March 2020), an episode for the first year of the pandemic (April 2020 to March 2021) and an episode for the second year of the pandemic (April 2021 to March 2022). The impact of the two pandemic episodes on rehabilitation success was assessed using multilevel mixed-effects generalised linear models. The difference between the questionnaire scores at the beginning and at the end of rehabilitation was chosen as the dependent variable. The two pandemic episodes, as well as possible confounding factors such as age, gender, duration of rehabilitation and the respective questionnaire scores at the beginning of rehabilitation were selected as fixed effects. The respective rehabilitation centre was specified as a random intercept.

In a first step, these multilevel mixed-effects generalised linear models were specified with a Gaussian family and identity link to illustrate the absolute effects of the pandemic on the respective questionnaire scales.

In a second step, identical multilevel mixed-effects generalised linear models were specified with a log-link in order to achieve better comparability between the questionnaire scales by illustrating the relative effects.

In a third step, the rigid categorisation into three episodes (pre-pandemic episode, first pandemic year and second pandemic year) was broken down and time (in quarters) was modelled as a non-linear variable. The non-linearity was implemented using restricted cubic splines with 5 knots and knot location based on Harrel’s recommended percentiles [23]. Once again, the remaining specification of the multilevel mixed-effects generalised linear model with a Gaussian family and identity link was retained.

In a final step, the SIMBO-C score was additionally included in the regression model. The SIMBO-C score was calculated at the beginning of rehabilitation and contains details on occupational problems and the job situation. Its inclusion should make it possible to distinguish between direct effects of the pandemic on the individual’s mental wellbeing and indirect effects via a complication of the occupational problem environment during the pandemic. In other words, we would like to know whether the success of rehabilitation is really affected by the pandemic, or whether it might rather be due to the changed occupational problems that the success of rehabilitation is peeled back by the pandemic.

No adjustment was made for multiple testing. Therefore, *p*-values should not be interpreted as confirmatory, but are descriptive in nature, and inferences drawn from the 95% confidence intervals may not be reproducible. Although the analysis is exploratory, the level of significance is defined as *p* < 0.05. All analyses were performed using Stata 17 (StataCorp, College Station, Texas, USA).

## Results

Between April 2019 and March 2022, a total of 18,388 patients from 7 rehabilitation centres could be included in the study. Of these patients, 82% submitted complete questionnaire data. The incomplete data submitted by the remaining 18% lacked, in particular, information on the length of the rehabilitation and the results of the questionnaire at the end of rehabilitation, which made an evaluation in terms of the effectiveness of the rehabilitation measure impossible. As shown in Figure S1, the proportion of incompletely submitted data decreased in the course of the pandemic, which indicates a lower dropout rate after the start of the pandemic.

The patient characteristics of those patients for whom complete information was available are shown in Table 1. There were no substantial shifts in the sex and age distribution of the patients. The average length of rehabilitation slightly increased in the first year of the pandemic but decreased again in the second year of the pandemic (*p* < 0.001).

**Table 1:** Baseline characteristics

		pre-pandemic episode			first year of the pandemic			second year of the pandemic			Total 3-year study time					
Cohort		(April 2019 to March 2020)			(April 2020 to March 2021)			(April 2021 to March 2022)			(April 2019 to March 2022)			*P*-value for differences between the three years		
N		**4952**			**5088**			**4978**			**15,018**					
Male sex		34.11%			34.63%			34.49%			34.41%			0.850		
Age, mean, SD		50.04	10.39		50.10	10.49		50.14	10.67		50.09	10.52		0.372		
Length of rehab, mean, SD		30.80	7.08		31.93	7.34		31.20	7.06		31.31	7.18		< 0.001		

The questionnaire scores at the beginning of the rehabilitation stay are shown in Table 2. In particular, the SIMBO-C score, a questionnaire that assesses occupational problems and the job situation showed substantial changes over the course of the pandemic (*p* < 0.001). A small increase in the questionnaire scores at the beginning of the rehabilitation measure compared to the pre-pandemic scores was seen for ‘psychological and somatoform disorders’, ‘psychological well-being’, ‘self-efficacy’ and ‘activity and participation’ as measured by the ICF questionnaire (all *p* < 0.05). This means that the psychosocial stress, documented at the beginning of the rehabilitation was greater during the pandemic than before the pandemic.

**Table 2:** Questionnaires at the beginning of the rehabilitation

		pre-pandemic episode			first year of the pandemic			second year of the pandemic			Total 3-year study time					
Cohort		(April 2019 to March 2020)			(April 2020 to March 2021)			(April 2021 to March 2022)			(April 2019 to March 2022)					
N		**4952**			**5088**			**4978**			**15,018**					
		**mean**	**SD**		**mean**	**SD**		**mean**	**SD**		**mean**	**SD**				
HEALTH-49: Psychological and somatoform disorders		52.33	10.42		52.96	10.62		53.52	10.56		52.94	10.54		< 0.001		
HEALTH-49: Psychological well-being		52.22	9.54		52.61	9.52		52.76	9.15		52.53	9.41		0.038		
HEALTH-49: Interactional problems		52.75	9.29		52.86	9.42		52.86	9.42		52.82	9.38		0.789		
HEALTH-49: Self-efficacy		51.86	9.96		52.17	10.09		52.34	9.93		52.13	10.00		0.037		
HEALTH-49: Activity and participation		53.91	9.51		54.17	9.54		54.37	9.50		54.15	9.52		0.070		
HEALTH-49: Social support		49.26	9.92		49.20	9.92		49.01	9.91		49.16	9.92		0.227		
HEALTH-49: Social stress		50.10	10.17		49.93	10.24		49.99	10.47		50.01	10.29		0.698		
ICF: Activity and participation		1.64	0.81		1.69	0.82		1.70	0.81		1.68	0.82		0.005		
SIMBO-C: Occupational problems and the job situation		36.73	27.71		40.64	28.11		40.73	27.04		39.38	27.69		< 0.001		
PAREMO: Change readiness		2.79	0.71		2.78	0.72		2.78	0.72		2.78	0.72		0.918		

The participants’ willingness to change, as measured by the respective domain of the PAREMO questionnaire, however, did not change over the course of the pandemic (*p* = 0.918).

**Fig. 1:**
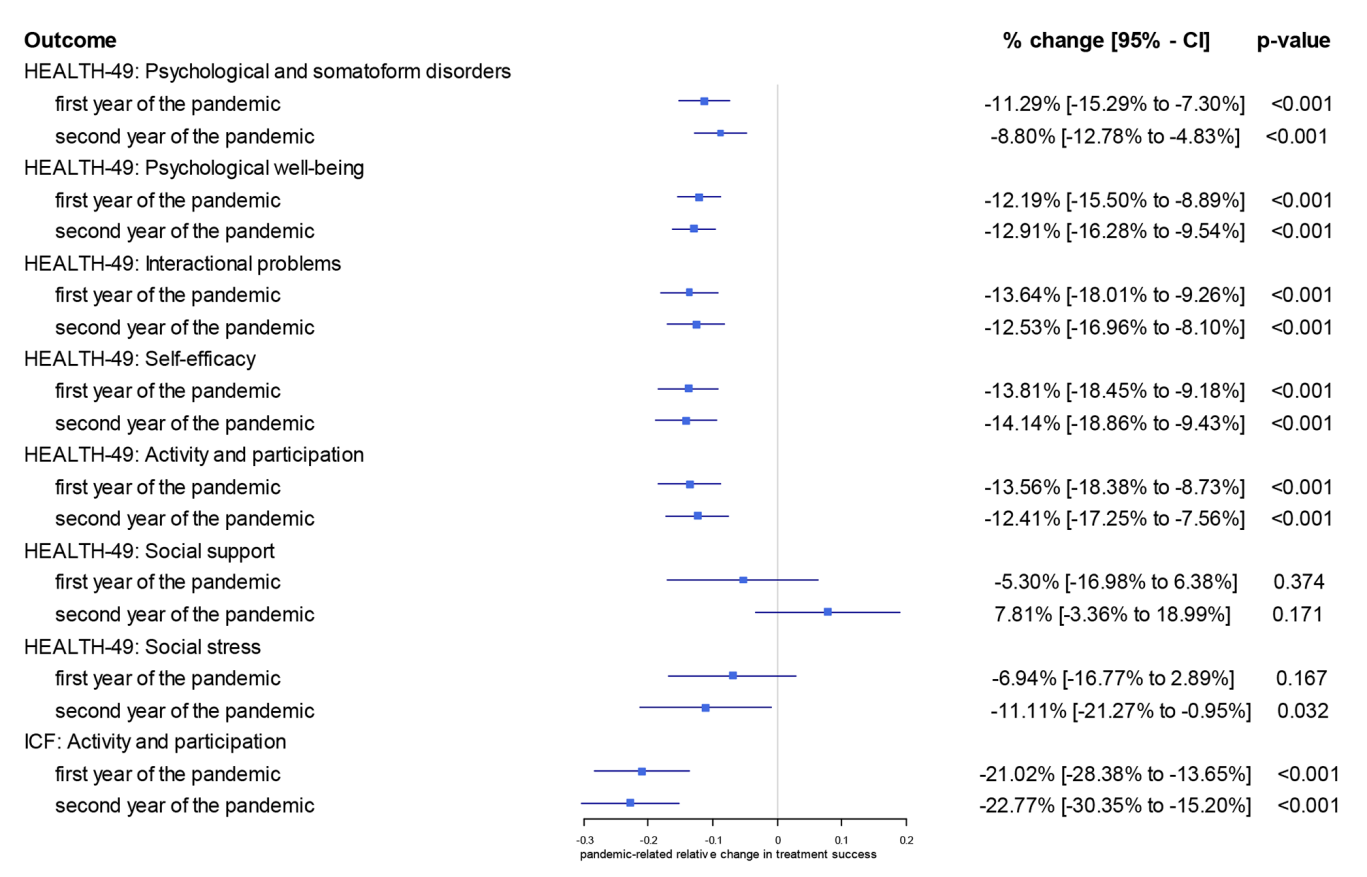
Relative effect of the pandemic on the effectiveness of the rehabilitation Effectiveness of the rehabilitation is defined as difference between the questionnaire scores at the beginning and at the end of rehabilitation. The pre-pandemic episode (April 2019 to March 2020) acted as reference and relative effects of the first year of the pandemic (April 2020 to March 2021) and the second year of the pandemic (April 2021 to March 2022) are shown using mixed-effects generalized linear models with a Gaussian family and log link. Due to the log link, the resulting coefficients may be interpreted as semi-elasticities. A semi-elasticity represents the percentage change in the dependent variable after a 1-fold absolute change in the independent variable. Possible confounding factors such as age, gender, duration of rehabilitation and the respective questionnaire scores at the beginning of rehabilitation were included as fixed effects and different rehabilitation centres were included as a random intercept

As shown in Table 3, there were significant differences in the effectiveness of the rehabilitation program between the three years for all dimensions analysed except ‘social support’. The results of the risk-adjusted regression models for the two years after the start of the pandemic confirm this picture and show a significant reduction in the effectiveness of the rehabilitation intervention in all dimensions analysed except ‘social support’. With regard to ‘psychological and somatoform disorders’, for example, there was an absolute reduction of the effectiveness of -0.666 (*p* < 0.001) in the first year of the pandemic and − 0.518 (*p* < 0.001) in the second year of the pandemic (See Table S1). As can be seen in Fig. 1, this translates into an 11.29% decrease in the effectiveness of the rehabilitation measure in the first year of the pandemic and an 8.8% decrease in the effectiveness of the rehabilitation measure in the second year of the pandemic. Similar reductions are shown for all dimensions analysed except ‘social support’. Interestingly, ‘social stress’ was more decreased in the second year of the pandemic. A significant recovery in the second year of the pandemic was shown for none of the dimensions. Interestingly, the pandemic-related reduction in the improvement of ‘activity and participation’ is significantly lower when this measure is assessed by the HEALTH-49 questionnaire compared to measurements by the ICF questionnaire.

**Table 3:** Effectiveness of the rehabilitation: Mean Difference between the questionnaire scores at the beginning and at the end of rehabilitation

		pre-pandemic episode			first year of the pandemic			second year of the pandemic			Total 3-year study time					
Cohort		(April 2019 to March 2020)			(April 2020 to March 2021)			(April 2021 to March 2022)			(April 2019 to March 2022)					
N		**4952**			**5088**			**4978**			**15,018**					
		**mean**	**SD**		**mean**	**SD**		**mean**	**SD**		**mean**	**SD**				
HEALTH-49: Psychological and somatoform disorders		6.65	7.37		6.21	7.32		6.41	7.29		6.42	7.33		0.026		
HEALTH-49: Psychological well-being		11.90	10.88		10.90	10.65		10.70	10.50		11.16	10.69		< 0.001		
HEALTH-49: Interactional problems		6.24	8.51		5.58	8.42		5.61	8.30		5.81	8.41		< 0.001		
HEALTH-49: Self-efficacy		6.77	9.62		6.05	9.69		5.89	9.63		6.23	9.65		< 0.001		
HEALTH-49: Activity and participation		7.16	9.91		6.35	9.82		6.38	9.88		6.63	9.88		< 0.001		
HEALTH-49: Social support		0.22	8.45		0.28	8.26		0.06	8.44		0.19	8.38		0.085		
HEALTH-49: Social stress		1.61	9.40		1.44	9.38		1.13	9.38		1.39	9.39		0.046		
ICF: Activity and participation		0.30	0.58		0.25	0.57		0.24	0.57		0.26	0.57		< 0.001		

**Fig. 2:**
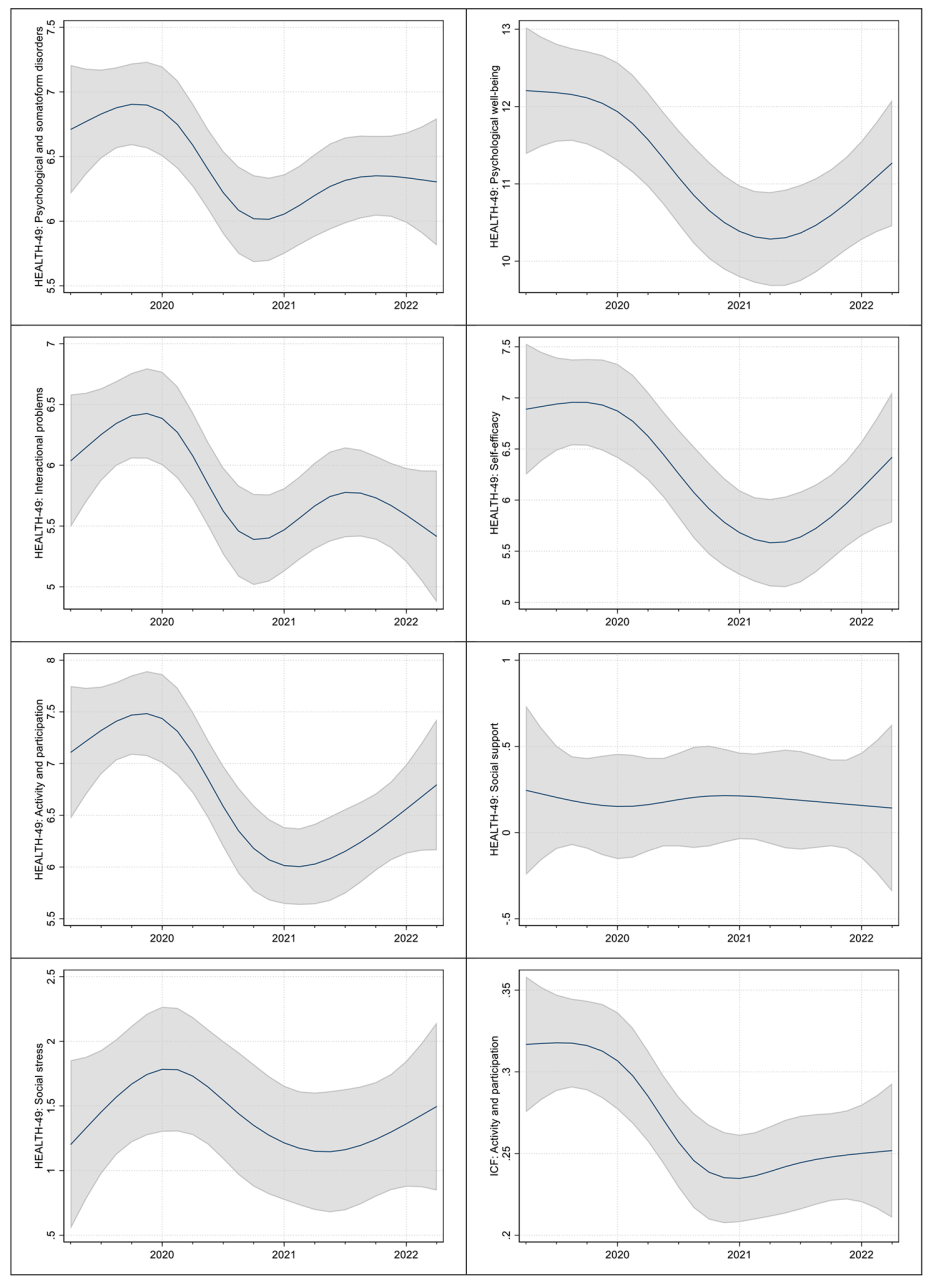
Absolute effect of the pandemic on the effectiveness of the rehabilitation Effectiveness of the rehabilitation is defined as difference between the questionnaire scores at the beginning and at the end of rehabilitation. For these vizualizations, the rigid categorisation into three episodes (pre-pandemic episode, first pandemic year and second pandemic year) was broken down and time (in quarters) was modelled as a non-linear variable. The non-linearity was implemented using restricted cubic splines with 5 knots and knot location based on Harrel’s recommended percentiles. Once again, the remaining specification of the multilevel mixed-effects generalised linear model with a Gaussian family and identity link was retained.

Figure 2 visualises the effectiveness measures over the 12 quarters. Here, too, there is no clear trend towards an improvement in the direction of the pre-pandemic situation.

Table S3 shows an additional adjustment for the SIMBO-C score for individual effectiveness measures. The background to this is to separate the direct effect of the pandemic from the indirect effect of workplace stress. As shown in Figure S2, the SIMBO-C score is substantially increased after the start of the pandemic. At the same time, there is a negative correlation between the SIMBO-C score and the effectiveness measures (here shown by ‘psychological and somatoform disorders’, See Figure S2). The adjustment for the SIMBO-C score results in a partial reduction of the effect of the pandemic on the effectiveness of the rehabilitation measure. With regard to ‘psychological and somatoform disorders’, for example, there was a reduction of the effectiveness of 11.3% (*p* < 0.001) in the first year of the pandemic. After further adjustment for the SIMBO-C score, this reduction of the effectiveness changes towards 7.5% (*p* < 0.001). Accordingly, we may interpret that roughly two thirds of the observed effect of the pandemic is directly related to the individual’s mental health and one third of the effect of the pandemic is indirectly related to the individual’s mental health via a complication of the occupational problem environment during the pandemic. A similar split between direct and indirect effects is seen for the second year of the pandemic and/or the other two dimensions ‘psychological well-being’ and ‘activity and participation’.

## Discussion

Overall, we found that the pandemic has substantially impaired the effectiveness of psychosomatic rehabilitation measures. This phenomenon can be observed across a wide range of psychosocial markers and even two years after the start of the pandemic there is no end to the limited effectiveness. Interestingly, the evaluations show that a division of the pandemic effect into direct effects (on the individual) and indirect effects (via further complication of the occupational problem environment) can be made and that the pandemic-related complications of the occupational problem environment are still highly prevalent more than two years after the start of the pandemic.

The most significantly impaired items in the HEALTH-49 questionnaire are psychological and somatoform disorders, psychological well-being and self-efficacy. It is understandable that those items are strongly compromised due to the introduction of restrictive measures, social distancing and the general uncertainty about the current situation and the future. However, it is remarkable that the items social support and social stress are not significantly deteriorated even though social contacts in person were barely possible because of social distancing regulations. Advanced digital technologies, which allow maintaining social contact could be a possible reason for the stability within these markers [24]. It is also conceivable that people are more eager to stay in close contact with families and friends in the face of a global emergency such as the pandemic, which could be an explanation for the persisting subjectively perceived social support [24].

Another interesting outcome is the impact of job- and workplace-related issues on the well-being of the patients. We found that only two thirds of the effect on mental health are caused directly by the pandemic itself, whereas the remaining third is indirectly impairing mental health via occupational difficulties. These complications are as well induced through the pandemic and the restrictions concerning work, schools and childcare [25]. Home schooling of children, short-time allowance and missing equipment for a smooth transition to remote work are only a few of the numerous problems employees had to face that are possible causes for increased stress regarding working environment and job security. Another study showed similar results concerning the impact of pre-rehabilitation scores on rehabilitation effectiveness during the pandemic [26]. They collected data from 1718 patients through an online survey between July 2020 and April 2021 using different questionnaires for the mental markers depression, anxiety, perceived stress and loneliness. Data collection took place at two different points of time: before the first day of their stay at the clinic and after completion of the program. The results show significant reduction in symptoms of all four test variables which is in line with the results of this study.

In contrast, an Austrian study could not find significant impairments in the effectiveness of rehabilitation programs during the pandemic [27]. In contrast to our study, they used the BSI-18 score and not the HEALTH-49 questionnaire to evaluate different psychological markers, but given that it is a reliable and valid self-assessment tool [28], the outcomes can be compared to our results. They however only compared two different points in time: before lockdown and closure of rehabilitation facilities and after reopening until December 2020.

Apart from the situation in psychosomatic rehabilitation facilities, it is important to notice the general situation for people with mental illnesses during the pandemic. Regulations and fear of infection made it difficult to seek support or continue the current therapy. A German study by Hoyer et al. investigates the utilization of mental health emergency service and travel activities during the weeks after the declaration of a pandemic by the WHO in week 11, 2020 [29]. They specified two different epochs (week 1–11 and week 12–15) both during 2019 and 2020 and compared these with regard to emergency service presentations. After introduction of the lockdown in week 12, emergency service presentation was only 69% compared to the same time period in 2019. At the same time, travelled kilometres and number of trips decreased significantly and remained at a continuing lower level.

A similar result was reported in a study concerning the provision of outpatient psychotherapy during the first weeks of lockdown in March 2020 compared to the months before [30]. Through an online survey, the researchers asked psychotherapists about their number of patients treated on average, including personal contact, via telephone and via internet and the changes after the introduction of legal regulations. The average number of treated patients dropped by 28%. Regarding the treatment in personal contact, Probst et al. found an average decrease of 81%, whereas treatment via telephone and via internet increased accordingly. Despite good compensation through technological alternatives, it was not manageable to continue providing the necessary support for all patients. This could be a consequence of missing familiarity with telepsychotherapy and other remote options to provide mental support. The effective use of web-based mental-health, however, is also associated with certain requirements such as the knowledge and technical equipment and the existence of private spaces to create a safeguarded atmosphere for consultation [31]. A recent systematic review identified several factors which contribute to the still low implementation of digital technologies in mental health care and made suggestions on how to overcome the barriers and to maximize their acceptability [32]. Although, web-based tools for mental health care are not free of disadvantages and limitations, this information could be used to make it easier for professionals to introduce web-based therapy into their usual daily practice so that the transition to telemedicine can be made smoothly in case of an emergency.

Our study has several limitations. First, the study design is retrospective and based on data from routine diagnostics. Consequently, coding errors are inevitable. Second, our analyses are based on self-reported data using questionnaires which may be biased by under- or overestimation of an item by the patients or lack of understanding of the specific question and thus inaccurate or false information. Third, for data protection reasons, the exact admission data of the patients was not available. Instead, only the respective admission quarters were transmitted. Given the local and temporal heterogeneity of regulations, it is therefore difficult to relate the values of the mental markers to the social situation at that same point of time. We do not have any information about the differences in terms of specific restrictions at that time regarding daily life as well as in the clinical setting. Thus, we cannot explore any restriction-related effects.

Fourth, we only collected data from seven different psychosomatic rehabilitation clinics located in three different regions in Germany (Baden-Württemberg, Thuringia and Saxony), all of them belonging to Celenus-Kliniken GmbH. Therefore it is uncertain whether our findings are sufficiently representative to draw conclusions for other similar facilities and regions.

Finally, when estimating the effect of the pandemic, adjusted differences in the respective outcomes can be interpreted as pandemic-related effects if all relevant parameters are used for risk adjustment [33]. Unfortunately, there can be no guarantee that all parameters of relevance are part of the model.

Nevertheless, a sample size of 15,018 may be considered a sufficient number of patients to explore the research questions. Another strength is the long period of time in which the data was collected. Most similar studies were published at the end of 2020, providing only figures collected during the first months of the pandemic and the first lockdown. With data from April 2019 until March 2022, we may cover several periods of varying restrictions, lockdown-like states and the second full lockdown. This allows us to look at the development of the impairment caused by the pandemic over time offering the potential of further insights in possible approaches in handling long-term and uncertain global crises.

Overall, our study shows that the Covid-19 pandemic has had a significant impact on the psychosomatic rehabilitation programs reducing the effectiveness of treatment not only for a short period of time but constantly until March 2022. The further development of the situation during the post-Covid phase still has to be investigated but our study clearly shows that there are still many improvements necessary to secure the protection and support for vulnerable groups during exceptional states such as the pandemic.

### Electronic supplementary material

Below is the link to the electronic supplementary material.

Supplementary Material 1

Supplementary Material 2

## Data Availability

The data that support the findings of this study are available from Celenus-Kliniken GmbH but restrictions apply to the availability of these data, which were used under license for the current study, and so are not publicly available. Data are however available from the authors upon reasonable request and with permission of Celenus-Kliniken GmbH.
