# Supplementary material for: The impact of the Covid-19 pandemic on the effectiveness of psychosomatic rehabilitation in Germany
Source: BMC Health Serv Res. 2024 Jun 11;24:719. doi: 10.1186/s12913-024-11170-1 (PMC11165732; doi:10.1186/s12913-024-11170-1)
Supplement: Supplementary file 1 — Supplementary Material 1 [file 12913_2024_11170_MOESM1_ESM.docx]

Figure S1: Proportion of patients with incompletely submitted data

Figure S2: Development of the SIMBO-C over time and the correlation between SIMBO-C values at admission and rehabilitation effectiveness

|  |  |
| --- | --- |
