# Supplementary material for: The impact of the Covid-19 pandemic on the effectiveness of psychosomatic rehabilitation in Germany
Source: BMC Health Serv Res. 2024 Jun 11;24:719. doi: 10.1186/s12913-024-11170-1 (PMC11165732; doi:10.1186/s12913-024-11170-1)
Supplement: Supplementary file 2 — Supplementary Material 2 [file 12913_2024_11170_MOESM2_ESM.docx]

Table S1: Absolute effect of the pandemic on the effectiveness of the rehabilitation

|  | HEALTH-49 | HEALTH-49 | HEALTH-49 | HEALTH-49 | HEALTH-49 | HEALTH-49 | HEALTH-49 | ICF |
| --- | --- | --- | --- | --- | --- | --- | --- | --- |
|  | Psychological and somatoform disorders | Psychological well-being | Interactional problems | Self-efficacy | Activity and participation | Social support | Social stress | Activity and participation |
|  |  |  |  |  |  |  |  |  |
| male sex | -0.958^***^ | -0.471^**^ | -0.035 | -0.202 | -1.202^***^ | -0.571^***^ | 0.223 | -0.047^***^ |
|  | [-1.194,-0.723] | [-0.811,-0.132] | [-0.291,0.222] | [-0.495,0.091] | [-1.514,-0.890] | [-0.824,-0.319] | [-0.065,0.512] | [-0.066,-0.028] |
|  |  |  |  |  |  |  |  |  |
| length of | 0.080^***^ | 0.114^***^ | 0.008 | 0.050^***^ | 0.057^***^ | -0.013 | -0.014 | 0.004^***^ |
| rehab | [0.064,0.096] | [0.091,0.136] | [-0.009,0.026] | [0.030,0.070] | [0.036,0.078] | [-0.030,0.004] | [-0.034,0.005] | [0.003,0.006] |
|  |  |  |  |  |  |  |  |  |
| score at | 0.213^***^ | 0.366^***^ | 0.407^***^ | 0.419^***^ | 0.381^***^ | 0.389^***^ | 0.399^***^ | 0.129^***^ |
| admission | [0.203,0.224] | [0.349,0.384] | [0.394,0.420] | [0.405,0.433] | [0.365,0.397] | [0.377,0.401] | [0.385,0.412] | [0.118,0.141] |
|  |  |  |  |  |  |  |  |  |
| age | -0.010^*^ | 0.037^***^ | -0.013^*^ | -0.025^***^ | -0.045^***^ | -0.013^*^ | -0.021^**^ | -0.004^***^ |
|  | [-0.020,0.001] | [0.022,0.053] | [-0.024,-0.001] | [-0.038,-0.012] | [-0.059,-0.031] | [-0.024,-0.001] | [-0.034,-0.008] | [-0.005,-0.003] |
|  |  |  |  |  |  |  |  |  |
| pre-pandemic |  | 0 (reference) |  |  |  |  |  |  |
|  |  |  |  |  |  |  |  |  |
| first year of | -0.666^***^ | -1.298^***^ | -0.729^***^ | -0.930^***^ | -0.971^***^ | 0.095 | -0.110 | -0.063^***^ |
| pandemic | [-0.937,-0.395] | [-1.691,-0.906] | [-1.024,-0.435] | [-1.269,-0.591] | [-1.332,-0.609] | [-0.197,0.388] | [-0.443,0.223] | [-0.085,-0.041] |
|  |  |  |  |  |  |  |  |  |
| second year | -0.518^***^ | -1.473^***^ | -0.637^***^ | -1.099^***^ | -0.987^***^ | -0.054 | -0.403^*^ | -0.068^***^ |
| pandemic | [-0.791,-0.246] | [-1.867,-1.078] | [-0.934,-0.341] | [-1.440,-0.757] | [-1.350,-0.623] | [-0.348,0.241] | [-0.739,-0.068] | [-0.090,-0.046] |
|  |  |  |  |  |  |  |  |  |
| constant | -5.196^***^ | -11.976^***^ | -14.738^***^ | -14.939^***^ | -11.201^***^ | -17.159^***^ | -17.128^***^ | 0.232^***^ |
|  | [-6.201,-4.191] | [-13.520,-10.433] | [-15.867,-13.608] | [-16.196,-13.683] | [-12.559,-9.843] | [-18.201,-16.117] | [-18.378,-15.878] | [0.161,0.302] |
| *N* | 15018 | 15006 | 14925 | 15012 | 14632 | 14900 | 14855 | 14851 |

95% confidence intervals in brackets

^*^ *p* < 0.10, ^**^ *p* < 0.01, ^***^ *p* < 0.001

Effectiveness of the rehabilitation is defined as difference between the questionnaire scores at the beginning and at the end of rehabilitation. The pre-pandemic episode (April 2019 to March 2020) acted as reference and absolute effects of the first year of the pandemic (April 2020 to March 2021) and the second year of the pandemic (April 2021 to March 2022) are shown using mixed-effects generalized linear models with a Gaussian family and identity link. Possible confounding factors such as age, gender, duration of rehabilitation and the respective questionnaire scores at the beginning of rehabilitation were included as fixed effects and different rehabilitation centres were included as a random intercept.

| Table S2: Relative effect of the pandemic on the effectiveness of the rehabilitation   \|  \| HEALTH-49 \| HEALTH-49 \| HEALTH-49 \| HEALTH-49 \| HEALTH-49 \| HEALTH-49 \| HEALTH-49 \| ICF \| \| --- \| --- \| --- \| --- \| --- \| --- \| --- \| --- \| --- \| \|  \| Psychological and somatoform disorders \| Psychological well-being \| Interactional problems \| Self-efficacy \| Activity and participation \| Social support \| Social stress \| Activity and participation \| \|  \|  \|  \|  \|  \|  \|  \|  \|  \| \| male sex \| -0.172^***^ \| -0.056^***^ \| -0.018 \| -0.042^*^ \| -0.195^***^ \| -0.221^***^ \| 0.004 \| -0.181^***^ \| \|  \| [-0.211,-0.134] \| [-0.086,-0.025] \| [-0.058,0.023] \| [-0.084,-0.001] \| [-0.241,-0.148] \| [-0.325,-0.118] \| [-0.090,0.097] \| [-0.251,-0.111] \| \|  \|  \|  \|  \|  \|  \|  \|  \|  \| \| length of \| 0.013^***^ \| 0.011^***^ \| 0.004^***^ \| 0.011^***^ \| 0.010^***^ \| 0.004 \| 0.006^*^ \| 0.018^***^ \| \| rehab \| [0.011,0.015] \| [0.009,0.013] \| [0.002,0.007] \| [0.008,0.014] \| [0.007,0.012] \| [-0.002,0.011] \| [0.000,0.011] \| [0.014,0.022] \| \|  \|  \|  \|  \|  \|  \|  \|  \|  \| \| score at \| 0.026^***^ \| 0.030^***^ \| 0.057^***^ \| 0.053^***^ \| 0.047^***^ \| 0.116^***^ \| 0.091^***^ \| 0.396^***^ \| \| admission \| [0.025,0.028] \| [0.029,0.032] \| [0.055,0.059] \| [0.051,0.056] \| [0.045,0.049] \| [0.111,0.121] \| [0.087,0.095] \| [0.360,0.432] \| \|  \|  \|  \|  \|  \|  \|  \|  \|  \| \| age \| -0.002^**^ \| 0.003^***^ \| -0.003^**^ \| -0.004^***^ \| -0.006^***^ \| -0.005^*^ \| -0.003^*^ \| -0.013^***^ \| \|  \| [-0.004,-0.001] \| [0.001,0.004] \| [-0.004,-0.001] \| [-0.006,-0.002] \| [-0.008,-0.005] \| [-0.009,-0.000] \| [-0.007,0.000] \| [-0.016,-0.011] \| \|  \| -0.172^***^ \| -0.056^***^ \| -0.018 \| -0.042^*^ \| -0.195^***^ \| -0.221^***^ \| 0.004 \| -0.181^***^ \| \| pre-pandemic \|  \| 0 (reference) \|  \|  \|  \|  \|  \|  \| \|  \|  \|  \|  \|  \|  \|  \|  \|  \| \| first year of \| -0.113^***^ \| -0.122^***^ \| -0.136^***^ \| -0.138^***^ \| -0.136^***^ \| -0.053 \| -0.069 \| -0.210^***^ \| \| pandemic \| [-0.153,-0.073] \| [-0.155,-0.089] \| [-0.180,-0.093] \| [-0.184,-0.092] \| [-0.184,-0.087] \| [-0.170,0.064] \| [-0.168,0.029] \| [-0.284,-0.137] \| \|  \|  \|  \|  \|  \|  \|  \|  \|  \| \| second year \| -0.088^***^ \| -0.129^***^ \| -0.125^***^ \| -0.141^***^ \| -0.124^***^ \| 0.078 \| -0.111^*^ \| -0.228^***^ \| \| pandemic \| [-0.128,-0.048] \| [-0.163,-0.095] \| [-0.170,-0.081] \| [-0.189,-0.094] \| [-0.173,-0.076] \| [-0.034,0.190] \| [-0.213,-0.009] \| [-0.303,-0.152] \| \|  \|  \|  \|  \|  \|  \|  \|  \|  \| \| *N* \| 15018 \| 15006 \| 14925 \| 15012 \| 14632 \| 14900 \| 14855 \| 14851 \|   95% confidence intervals in brackets  ^*^ *p* < 0.10, ^**^ *p* < 0.01, ^***^ *p* < 0.001 |
| --- | --- | --- | --- | --- | --- | --- | --- | --- | --- | --- | --- | --- | --- | --- | --- | --- | --- | --- | --- | --- | --- | --- | --- | --- | --- | --- | --- | --- | --- | --- | --- | --- | --- | --- | --- | --- | --- | --- | --- | --- | --- | --- | --- | --- | --- | --- | --- | --- | --- | --- | --- | --- | --- | --- | --- | --- | --- | --- | --- | --- | --- | --- | --- | --- | --- | --- | --- | --- | --- | --- | --- | --- | --- | --- | --- | --- | --- | --- | --- | --- | --- | --- | --- | --- | --- | --- | --- | --- | --- | --- | --- | --- | --- | --- | --- | --- | --- | --- | --- | --- | --- | --- | --- | --- | --- | --- | --- | --- | --- | --- | --- | --- | --- | --- | --- | --- | --- | --- | --- | --- | --- | --- | --- | --- | --- | --- | --- | --- | --- | --- | --- | --- | --- | --- | --- | --- | --- | --- | --- | --- | --- | --- | --- | --- | --- | --- | --- | --- | --- | --- | --- | --- | --- | --- | --- | --- | --- | --- | --- | --- | --- | --- | --- | --- | --- | --- | --- | --- | --- | --- | --- | --- | --- | --- | --- | --- | --- | --- | --- | --- | --- | --- | --- | --- | --- | --- | --- | --- | --- | --- | --- | --- | --- | --- | --- | --- | --- | --- | --- | --- | --- | --- | --- | --- | --- | --- | --- | --- | --- | --- | --- | --- | --- | --- | --- | --- |
| Effectiveness of the rehabilitation is defined as difference between the questionnaire scores at the beginning and at the end of rehabilitation. The pre-pandemic episode (April 2019 to March 2020) acted as reference and relative effects of the first year of the pandemic (April 2020 to March 2021) and the second year of the pandemic (April 2021 to March 2022) are shown using mixed-effects generalized linear models with a Gaussian family and log link. Due to the log link, the resulting coefficients may be interpreted as semi-elasticities. A semi-elasticity represents the percentage change in the dependent variable after a 1-fold absolute change in the independent variable. Possible confounding factors such as age, gender, duration of rehabilitation and the respective questionnaire scores at the beginning of rehabilitation were included as fixed effects and different rehabilitation centres were included as a random intercept. |

| Table S3: Relative effect of the pandemic on the effectiveness of the rehabilitation: A look at the SIMBO-C score   \|  \| HEALTH-49 \| HEALTH-49 \| HEALTH-49 \| HEALTH-49 \| ICF \| ICF \| \| --- \| --- \| --- \| --- \| --- \| --- \| --- \| \|  \| Psychological and somatoform disorders \| Psychological and somatoform disorders \| Psychological well-being \| Psychological well-being \| Activity and participation \| Activity and participation \| \|  \|  \|  \|  \|  \|  \|  \| \| male sex \| -0.172^***^ \| -0.133^***^ \| -0.056^***^ \| -0.003 \| -0.181^***^ \| -0.127^***^ \| \|  \| [-0.211,-0.134] \| [-0.170,-0.095] \| [-0.086,-0.025] \| [-0.030,0.025] \| [-0.251,-0.111] \| [-0.192,-0.062] \| \|  \|  \|  \|  \|  \|  \|  \| \| length of \| 0.013^***^ \| 0.013^***^ \| 0.011^***^ \| 0.011^***^ \| 0.018^***^ \| 0.017^***^ \| \| rehab \| [0.011,0.015] \| [0.011,0.015] \| [0.009,0.013] \| [0.009,0.012] \| [0.014,0.022] \| [0.013,0.020] \| \|  \|  \|  \|  \|  \|  \|  \| \| score at \| -0.002^**^ \| -0.004^***^ \| 0.003^***^ \| 0.000 \| -0.013^***^ \| -0.015^***^ \| \| admission \| [-0.004,-0.001] \| [-0.006,-0.003] \| [0.001,0.004] \| [-0.001,0.001] \| [-0.016,-0.011] \| [-0.018,-0.013] \| \|  \|  \|  \|  \|  \|  \|  \| \| age \| -0.002^**^ \| 0.003^***^ \| -0.003^**^ \| -0.004^***^ \| -0.003^*^ \| -0.013^***^ \| \|  \| [-0.004,-0.001] \| [0.001,0.004] \| [-0.004,-0.001] \| [-0.006,-0.002] \| [-0.007,0.000] \| [-0.016,-0.011] \| \|  \| -0.172^***^ \| -0.056^***^ \| -0.018 \| -0.042^*^ \| 0.004 \| -0.181^***^ \| \| pre-pandemic \|  \| 0 (reference) \|  \|  \|  \|  \| \|  \|  \|  \|  \|  \|  \|  \| \| first year of \| -0.113^***^ \| -0.075^***^ \| -0.122^***^ \| -0.073^***^ \| -0.210^***^ \| -0.127^***^ \| \| pandemic \| [-0.153,-0.073] \| [-0.114,-0.037] \| [-0.155,-0.089] \| [-0.103,-0.042] \| [-0.284,-0.137] \| [-0.196,-0.059] \| \|  \|  \|  \|  \|  \|  \|  \| \| second year \| -0.088^***^ \| -0.061^**^ \| -0.129^***^ \| -0.079^***^ \| -0.228^***^ \| -0.162^***^ \| \| pandemic \| [-0.128,-0.048] \| [-0.100,-0.022] \| [-0.163,-0.095] \| [-0.110,-0.047] \| [-0.303,-0.152] \| [-0.233,-0.091] \| \|  \|  \|  \|  \|  \|  \|  \| \| SIMBO-C \|  \| -0.168^***^ \|  \| -0.222^***^ \|  \| -0.309^***^ \| \|  \|  \| [-0.182,-0.154] \|  \| [-0.233,-0.211] \|  \| [-0.335,-0.283] \| \|  \|  \|  \|  \|  \|  \|  \| \| *N* \| 15018 \| 15006 \| 14925 \| 15012 \| 14855 \| 14851 \|   95% confidence intervals in brackets  * p < 0.10, ** p < 0.01, *** p < 0.001 |
| --- | --- | --- | --- | --- | --- | --- | --- | --- | --- | --- | --- | --- | --- | --- | --- | --- | --- | --- | --- | --- | --- | --- | --- | --- | --- | --- | --- | --- | --- | --- | --- | --- | --- | --- | --- | --- | --- | --- | --- | --- | --- | --- | --- | --- | --- | --- | --- | --- | --- | --- | --- | --- | --- | --- | --- | --- | --- | --- | --- | --- | --- | --- | --- | --- | --- | --- | --- | --- | --- | --- | --- | --- | --- | --- | --- | --- | --- | --- | --- | --- | --- | --- | --- | --- | --- | --- | --- | --- | --- | --- | --- | --- | --- | --- | --- | --- | --- | --- | --- | --- | --- | --- | --- | --- | --- | --- | --- | --- | --- | --- | --- | --- | --- | --- | --- | --- | --- | --- | --- | --- | --- | --- | --- | --- | --- | --- | --- | --- | --- | --- | --- | --- | --- | --- | --- | --- | --- | --- | --- | --- | --- | --- | --- | --- | --- | --- | --- | --- | --- | --- | --- | --- | --- | --- | --- | --- | --- | --- | --- | --- | --- | --- | --- | --- | --- | --- | --- | --- | --- | --- | --- | --- | --- | --- | --- | --- | --- | --- | --- | --- | --- | --- | --- | --- | --- | --- | --- | --- | --- |

Effectiveness of the rehabilitation is defined as difference between the questionnaire scores at the beginning and at the end of rehabilitation. The pre-pandemic episode (April 2019 to March 2020) acted as reference and relative effects of the first year of the pandemic (April 2020 to March 2021) and the second year of the pandemic (April 2021 to March 2022) are shown using mixed-effects generalized linear models with a Gaussian family and log link. Due to the log link, the resulting coefficients may be interpreted as semi-elasticities. A semi-elasticity represents the percentage change in the dependent variable after a 1-fold absolute change in the independent variable. Possible confounding factors such as age, gender, duration of rehabilitation and the respective questionnaire scores at the beginning of rehabilitation were included as fixed effects and different rehabilitation centres were included as a random intercept.
